# Endothelial Cells Stably Infected with Recombinant Kaposi’s Sarcoma-Associated Herpesvirus Display Distinct Viscoelastic and Morphological Properties

**DOI:** 10.1007/s12195-025-00848-z

**Published:** 2025-04-18

**Authors:** Majahonkhe M. Shabangu, Melissa J. Blumenthal, Danielle T. Sass, Dirk M. Lang, Georgia Schafer, Thomas Franz

**Affiliations:** 1 Biomedical Engineering Research Centre, Division of Biomedical Engineering, Department of Human Biology, Faculty of Health Sciences, University of Cape Town, Observatory, Cape Town, 7925 South Africa; 2 International Centre for Genetic Engineering and Biotechnology, Observatory, Cape Town, 7925 South Africa; 3 Institute of Infectious Disease and Molecular Medicine, Faculty of Health Sciences, University of Cape Town, Observatory, Cape Town, 7925 South Africa; 4 Division of Medical Biochemistry, Department of Integrative Biomedical Sciences, Faculty of Health Sciences, University of Cape Town, Observatory, Cape Town, 7925 South Africa; 5 Division of Physiological Sciences, Department of Human Biology, Faculty of Health Sciences, University of Cape Town, Observatory, Cape Town, 7925 South Africa; 6 Bioengineering Science Research Group, Faculty of Engineering and Physical Sciences, University of Southampton, Southampton, UK

**Keywords:** Cell mechanics, KSHV, Mechanotyping, Passive microrheology, Vascular, Lymphatic

## Abstract

**Purpose:**

Kaposi’s sarcoma-associated herpesvirus (KSHV) is a γ-herpesvirus that has a tropism for endothelial cells and leads to the development of Kaposi’s sarcoma, especially in people living with HIV. The present study aimed to quantify morphological and mechanical changes in endothelial cells after infection with KSHV to assess their potential as diagnostic and therapeutic markers.

**Methods:**

Vascular (HuARLT2) and lymphatic endothelial cells (LEC) were infected with recombinant KSHV (rKSHV) by spinoculation, establishing stable infections (HuARLT2-rKSHV and LEC-rKSHV). Cellular changes were assessed using mitochondria-tracking microrheology and morphometric analysis.

**Results:**

rKSHV infection increased cellular deformability, indicated by higher mitochondrial mean squared displacement (MSD) for short lag times. Specifically, MSD at τ = 0.19 s was 49.4% and 42.2% higher in HuARLT2-rKSHV and LEC-rKSHV, respectively, compared to uninfected controls. There were 23.9% and 36.7% decreases in the MSD power law exponents for HuARLT2-rKSHV and LEC-rKSHV, respectively, indicating increased cytosolic viscosity associated with rKSHV infection. Infected cells displayed a marked spindloid phenotype with an increase in aspect ratio (29.7%) and decreases in roundness (26.1%) and circularity (25.7%) in HuARLT2-rKSHV, with similar changes observed in LEC-rKSHV.

**Conclusions:**

The quantification of distinct KSHV-induced morpho-mechanical changes in endothelial cells demonstrates the potential of these changes as diagnostic markers and therapeutic targets.

## Introduction

The Kaposi’s sarcoma-associated herpesvirus (KSHV), also known as human gammaherpesvirus 8 (HHV-8), is the aetiological agent of Kaposi’s sarcoma (KS), a malignancy that affects the endothelium [1]. KS presents primarily as cutaneous lesions composed of proliferative spindle cells of endothelial origin, often occurring in the immunocompromised such as people living with HIV (PLWH) and organ transplant recipients [2]. KSHV infection in endothelial cells (ECs) can follow latent or lytic pathways. Latent infection, the default pathway, involves limited viral gene expression with the viral genome maintained as an episome in infected ECs. Spontaneous lytic reactivation in a subset of infected cells leads to extensive viral gene expression, new virion production, and infected cell death [3]. KSHV induces significant genetic and phenotypic changes in ECs, notably reorganisation of the cytoplasmic actin cytoskeleton by the viral latency-associated v-FLIP gene, which leads to the hallmark spindle-shaped cell morphology of KS-associated cells [4].

Early diagnosis of KS is critical for a favourable prognosis. However, a confirmed KS diagnosis is resource-intensive and not always attainable in resource-limited settings that, for example, may have a shortage of experienced pathologists who can discern its various subtleties [5, 6]. The resource-intensive diagnostic process involves histopathological identification of spindle cells from suspect lesion biopsies, which is usually confirmed by a positive latency-associated nuclear antigen (LANA) stain test. Although consistent use of combination antiretroviral therapy has led to regression of KS lesions in PLWH, poor outcomes persist in sub-Saharan Africa, exacerbated in part by high KSHV and HIV seroprevalence [7]. The recent emergence of KS in virally suppressed PLWH underscores the need for further investigation of the pathobiology and treatment of KS [8].

During KSHV infection of target ECs, the cytoskeleton is implicated in viral entry and post-infection transcriptional dysregulation events, including endothelial-to-mesenchymal transition (EndMT) [9, 10]. KSHV-induced actin reorganisation mediates the hallmark spindle morphology of KSHV-infected ECs. Given the central role of the actin cytoskeleton in modulating cell mechanics, the actin reorganisation indicates a possible association of KSHV infection with changes in intracellular viscoelastic properties [11]. Studies have demonstrated notable changes in cellular and organelle mechanical properties brought on by the infection of cells in vitro with herpesvirus and adenovirus [12, 13] and rubella virus [14]. If precisely defined, these intracellular stiffness properties can contribute to improved diagnosis and treatment of diseases associated with infections by these viruses. In the case of KS, the cell mechanical phenotype can serve as an alternative to identify and target the otherwise heterogeneous KS lesion cells.

The current study aimed to determine changes in the mechanical and morphological properties of ECs associated with their infection with KSHV as a precursor for KS tumorigenesis. The morphological and intracellular viscoelastic properties concomitant with latent recombinant KSHV infection of immortalised EC lines of vascular (HuARLT2) and lymphatic (LEC) origin were evaluated in vitro. Given the ongoing search for the true cellular origin of KS [5] with KSHV infection inducing lymphatic traits in vascular ECs and vascular characteristics in lymphatic ECs [15, 16] , examining both cell types in the current study was crucial. This approach ensured that both endothelial origins, which are strongly targeted by KSHV, were investigated.

## Materials and Methods

### Overview

**Fig. 1:**
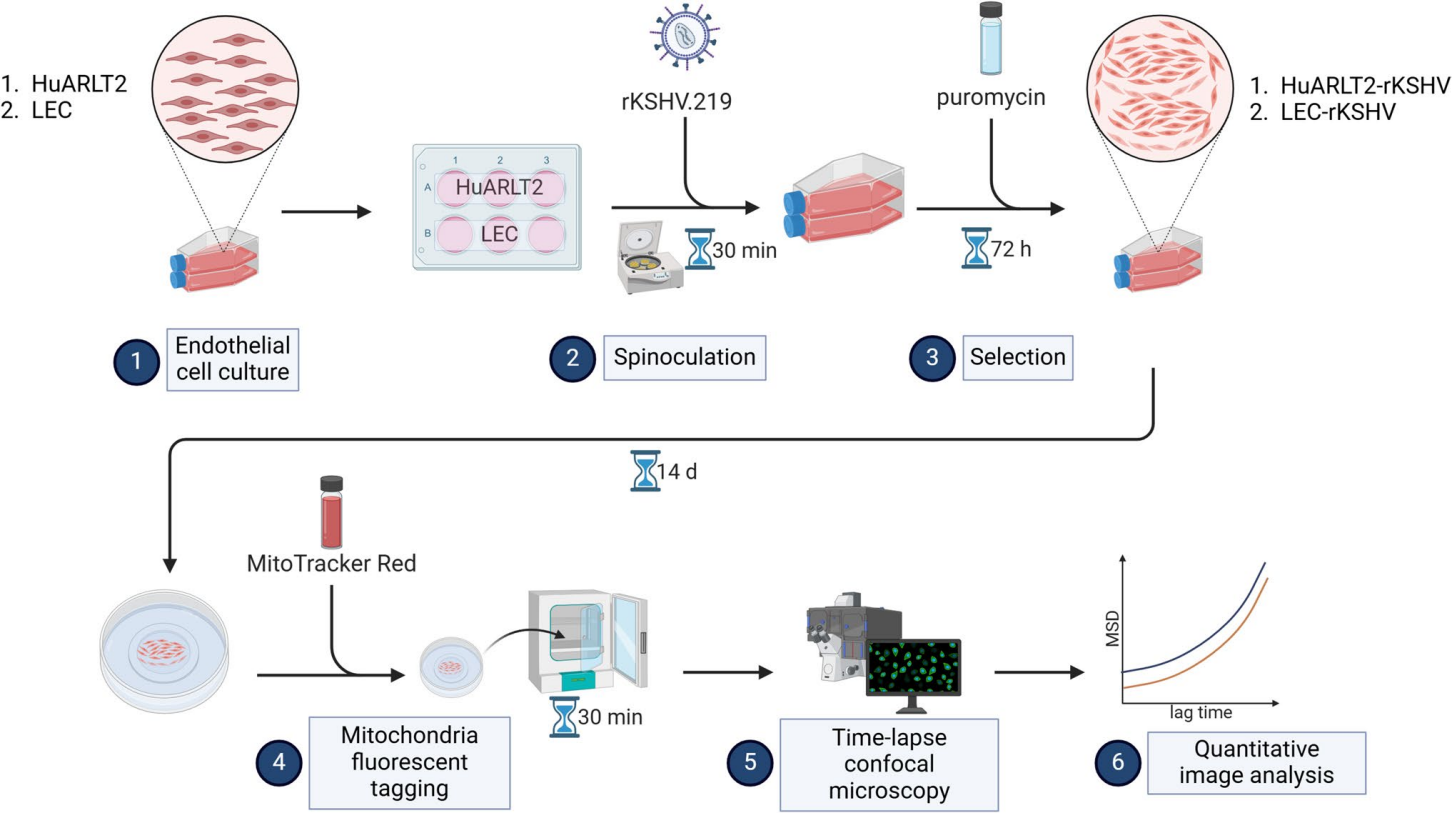
Methodology workflow. High-level illustration of sequential steps (1–6) of the approach used. (Created with BioRender.com)

The methodological approach used in the current study is illustrated in Fig. 1 and described in the subsequent sections.

### Cell Lines

As previously described [17], BJAB-rKSHV.219 were established from BJAB cells (DSMZ No.: ACC 757), a human Burkitt lymphoma-derived cell line, via stable infection with a recombinant Kaposi’s sarcoma-associated herpesvirus (rKSHV.219). rKSHV.219, isolated from JSC-1 primary effusion lymphoma cells, expresses the green fluorescent protein (GFP) to indicate latency and the red fluorescent protein (RFP) to indicate lytic infection driven by cellular EF-1α and KSHV lytic PAN promoters, respectively, and contains a gene for puromycin resistance as a selectable marker [17, 18].

HuARLT2 cells are a conditionally-immortalised human umbilical vein endothelial cell line (HUVEC) derivative established via the expression of doxycycline-inducible transgenes simian virus 40 (SV40) large T antigen (TAg) and human telomerase reverse transcriptase (hTert) [19].

LECs are a telomerase-immortalised human lymphatic endothelial cell (LEC) line [20, 21].

Both HuARLT2 and LEC cell lines are well-established models for investigating KS mechanisms in vitro [22, 23].

HEK-293T (DSMZ No.: ACC 305) cells are derived from human embryonic kidney 293 cells (HEK-293, Cellonex, C293-C) by stably expressing the transgenes SV40 and Tag.

BJAB-rKSHV.219, HuARLT2 and HEK-293T cell lines were provided by Professor Thomas Schulz (MHH, Hannover, Germany). The LEC cell line was a gift from Dr Frank Neipel (Institute of Clinical and Molecular Virology, University Clinic, Erlangen, Germany).

### Cell Culture

BJAB-rKSHV.219 cells were maintained in Roswell Park Memorial Institute (RPMI; Sigma-Aldrich, South Africa) 1640 medium supplemented with 20% foetal bovine serum (FBS; Sigma-Aldrich) in the presence of 4.2 μg/ml puromycin [23].

HuARLT2 cells were grown and maintained in EGM-2MV BulletKit growth medium (Endothelial cell basal medium supplemented with SingleQuots; Lonza) at 37 °C with 5% CO_2_ in the presence of 1 μg/ml doxycycline (Sigma-Aldrich).

LECs were maintained in EGM-2MV medium only.

HEK-293T cells were grown and maintained in Dulbecco’s Modified Eagle Medium (DMEM; Gibco) supplemented with 10% FBS.

For sub-culturing adherent cell lines and plating for downstream experiments, accutase (Biowest) was used to detach endothelial cell lines (HuARLT2 and LEC), and trypsin was used for HEK-293T cells. All cell quantifications were performed using the Countess Automated Cell Counter (Invitrogen).

### Recombinant KSHV (rKSHV.219) Production

For recombinant KSHV production, latently and stably infected BJAB cells (BJAB-rKSHV.219) were used as described previously [17]. Briefly, BJAB-rKSHV.219 cells were cultured in 500 ml reusable spinner flasks (Beckman Coulter) at a density of 6 × 10^5^ cells/ml in 400 ml of glutamate-free RPMI supplemented with 20% FBS under constant agitation of 60 rpm for 4–5 days and in the presence of 2.5 μg/ml anti-human IgM antibody (Sigma-Aldrich) to induce the KSHV lytic cycle. After 5 days, the contents of the spinner flask were subjected to low-speed centrifugation in 50 ml Falcon tubes to remove cell debris and to collect supernatants in sterilised 250 ml centrifuge bottles (Corning). The virus was pelleted from supernatants via centrifugation at 14,000 rpm for 6 h at 4 °C using a Beckman J2-21 high-speed ultracentrifuge fitted with a JA14 rotor (Beckman Coulter Inc., Fullerton, CA, USA). After carefully removing supernatants, virus pellets were re-suspended in 1 ml of EGM-2MV and stored at 4 °C for use within 4 weeks.

### Determination of rKSHV.219 Titres

HEK-293T cells cultured in DMEM supplemented with 10% FBS and plated on a 96-well plate at a density of 3 × 10^5^ cells/well were infected with rKSHV.219 using the serial dilutions method and spinoculation, where plates were centrifuged at 450×*g* for 30 min at 30 °C. Three days post-infection, GFP-positive cells in selected wells were manually counted using fluorescent microscope imaging and the virus titre was determined in infectious units per ml (IU/ml).

### Infection of Endothelial Cell Lines with rKSHV.219

HuARLT2s and LECs were infected with rKSHV.219 by spinoculation, as described before [23]. Briefly, HuARLT2 cells and LECs were plated on a 6-well plate at a density of 5 × 10^5^ cells/well and subjected to rKSHV.219 infection via low-speed centrifugation of 450×*g* for 30 min at 30 °C at multiplicities of infection (MOI) of 0.1 and 1, respectively, in the presence of 10 μg/ml polybrene (Sigma). EGM-2MV media containing 10 μg/ml polybrene was used for mock infections. Inoculant media was replaced with normal EGM-2MV 8 h after spinoculation. After 3 days, normal media was replaced with selection media comprising EGM-2MV media containing 5 μg/ml puromycin and 1 μg/ml doxycycline for infected HuARLT2 cells and 0.25 μg/ml puromycin only for infected LECs. Infected cells were maintained under selection for at least 2 weeks before particle tracking microrheology analysis. Latent infection in HuARLT2-rKSHV.219 and LEC-rKSHV.219 was confirmed qualitatively through GFP expression observed using a confocal fluorescent microscope (ZEISS LSM 880, Germany), controlled by Zen Black software version 2.3 (Carl Zeiss) under the emission filter of 488 nm.

### Mitochondria-Tracking Microrheology

Mitochondria-tracking microrheology (MTM) was used to mechanically characterise the intracellular environment of cells by measuring and analysing the mean squared displacement (MSD) of individual mitochondria over time as described before [24].

Cells were seeded on pre-equilibrated, uncoated, 4-compartment glass-bottom viewing dishes (µ-Dish 35 mm Quad–Ibidi, GmbH, Gräfelfing, Germany) suitable for confocal microscopy at a seeding density of 1.2 × 10^3^ cells/ml and kept overnight at 37 °C with 5% CO_2_. EGM-2MV media was replaced with 100 nM of fluorescent mitochondria dye (MitoTracker Red FM) in EGM-2MV. Each quad was filled with up to 300 μl of staining media, and the viewing dish was incubated away from light at 37 °C with 5% CO_2_ for 30 min. The staining medium was removed, and cells were rinsed with EGM-2MV twice before microscopic live cell imaging.

All imaging was performed in a 37 °C, 5% CO_2_ environmental chamber. For time-lapse imaging of live cells, between eight and 12 non-overlapping fields of view (FOV) were acquired for each condition (i.e., viewing dish quad) using an inverted laser scanning confocal microscope equipped with a monochrome CCD camera (ZEISS LSM 880 with Airyscan, Germany), with a 63× oil immersion lens with 1.4 NA for 100 s at a frame rate of 10 Hz. An emission band pass filter of 585–640 nm was used. To enhance the signal-to-noise ratio (SNR) and achieve a frame rate of at least 10 Hz, a 2 × 2 binning mode was used for a final spatial resolution of 0.33 μm/pixel. One thousand frames of 688 × 520 pixels were acquired for each FOV. At least two non-interacting cells from each FOV with a minimum of 50 identifiable punctate mitochondria were selected for particle tracking and analysis. Ten fluorescent images per experimental condition were collected using the same imaging set-up to identify latently infected GFP-expressing cells for particle tracking analysis.

Post-processing of time-lapse image series (x, y, t) was performed based on the approach described by Xu [25]. Briefly, image stacks were pre-processed using FIJI [26] via the following pipeline: (i) cropping single cells (ii) conversion to 8-bit tiff stack (iii) histogram matching of all frames to the first frame to correct for bleaching (iv) background subtraction using a 40-pixel rolling radius (v) 2D deconvolution using 25 iterations to deblur the stack (vi) Gaussian blur filter with varying radii adjusted according to the SNR of each series.

Thereafter, the plugin TrackMate [27] was used to segment image stacks, capture the spatial coordinates of near-punctate mitochondria, and generate and encode mitochondrial tracks into XML files. The Python-based Trackpy library version 0.5.0 [28] was used to process XML track files into mean squared displacement (MSD) curves and associated MSD power law exponents (α-values). The MSD was reported for individual mitochondrion as time-averaged MSD (TAMSD), for multiple mitochondria of a single cell as ensemble-averaged MSD (EAMSD), and for multiple cells within the same group as the mean ensemble-averaged MSD (or bulk MSD). The EAMSD was determined according to:

1 $$\overline{{\left\langle {\Delta \text{r}^{2} \left( {\text{T}, \uptau } \right)} \right\rangle }} = \frac{1}{{\text{T}{ - }\uptau }}\mathop \sum \limits_{0}^{{\text{T}{ - }\uptau }} |\text{X}\left( {\text{t} + \uptau } \right){ - }\text{X}( \text{t})|^{2} ,$$

where $\Delta$r^2^ is the MSD, T is the total acquisition time, t is the iterative time point, τ is the lag time, that is, the time interval between consecutive image pairs used for displacement calculations, X(t) and X(t + τ) are the position vectors in 2D space of mitochondrion at times t and t + τ, respectively. The angle brackets 〈〉 represent the TAMSD, and the overline ‾ represents the EAMSD [29]. The MSD varies nonlinearly with the lag time following a power law relationship for viscoelastic materials such as the intracellular environment, that is, $\left\langle {\Delta {\text{r}}^{2} ({\text{T}},\uptau )} \right\rangle \simeq \uptau ^{\upalpha }$. The MSD power law exponent, α, is a derivative quantitative parameter based on the EAMSD. It describes the diffusive mode of tracked particles and is calculated as the logarithmic gradient of the MSD-$\tau$ curve [30]:

2 $$\upalpha = \frac{{{\text{d}}\,\ln \left( {\left\langle {\Delta {\text{r}}^{2} ({\text{T}},\uptau )} \right\rangle } \right)}}{{{\text{d}}\,\ln (\uptau )}}$$

In relation to cellular viscoelasticity, MSD amplitude as a function of lag time serves as a proxy for bulk deformability while its power law exponent reflects cytosolic viscosity. For example, larger MSD amplitude suggests a less dense or more flexible cytoskeletal network indicative of increased cellular deformability. A lower power law signifies restricted mitochondrial diffusion, pointing to a more viscous or crowded cytosolic environment. Taken together, these parameters reflect the biphasic material model of the cytoplasm and attempt to characterise its complex rheology [31].

For extremely jagged EAMSD curves, indicative of highly dynamic mitochondrial fluctuations, the Savitzky–Golay filter with adaptive window size (W_SG_ = 7) and polynomial order (P_SG_ = 3) was applied. This adaptive approach ensured that the coefficient of determination (R^2^) for all power law fits within the lag time range of 0–2 s was R^2^ ≥ 0.7. Power law fitting was limited to this range to focus mainly on passive mitochondrial fluctuations which are representative of Brownian motion. This allows for the extraction of reliable viscoelastic properties of the cytoplasm as reflected in the MSD and its power law exponent. At longer lag times, directed motion and ATP-dependent processes can confound the interpretation of extracted parameters [25].

### Analysis of Cell Morphology

**Fig. 2:**
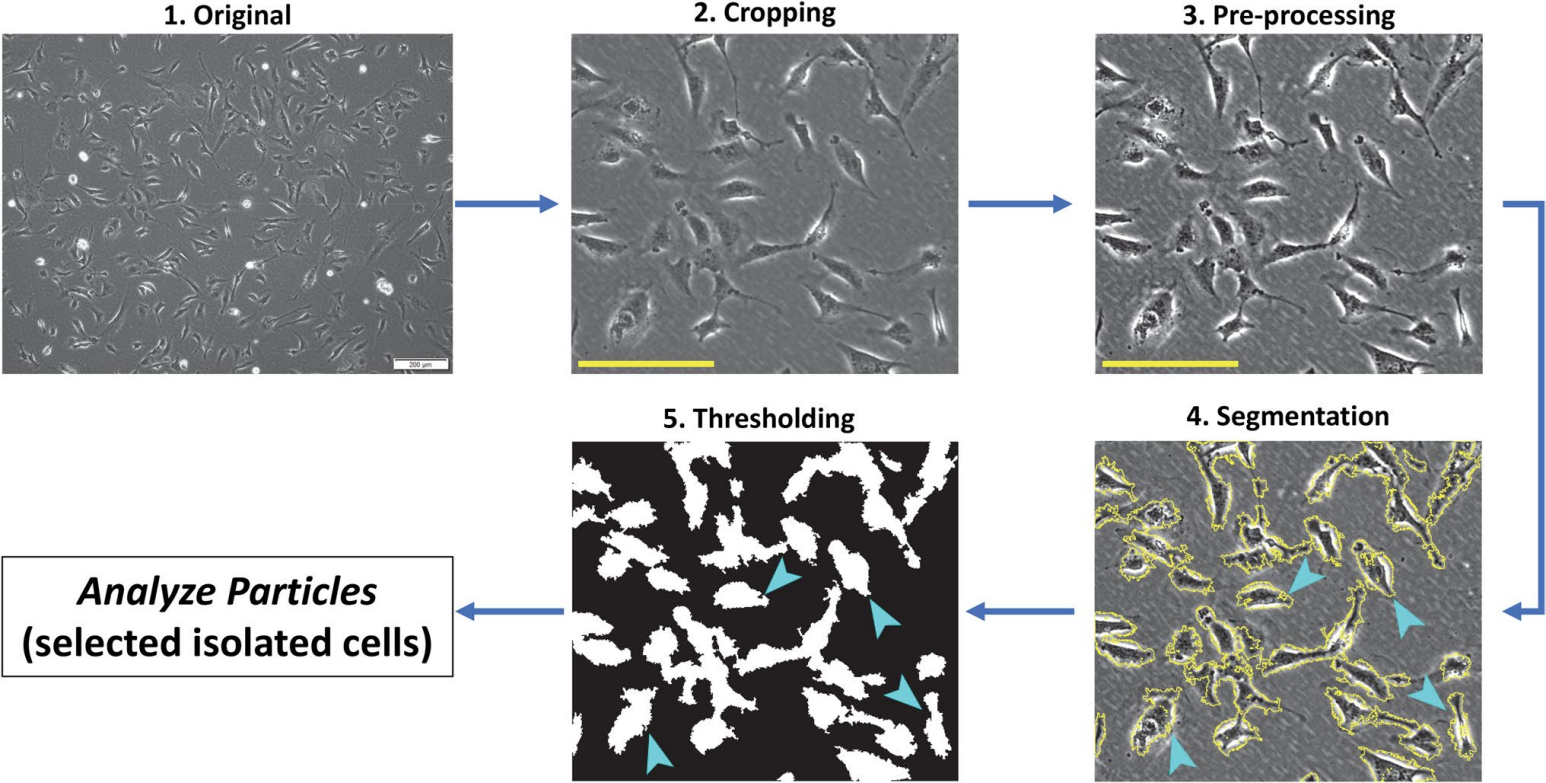
Image processing workflow for morphological analysis using FIJI and PHANTAST. Arrowheads indicate isolated cells earmarked for analysis. Scale bar = 500 mm.

Phase contrast and fluorescence images of cells were captured using the Olympus IX81 inverted microscope (Olympus Corporation, Tokyo, Japan) at ×10 magnification, controlled by Olympus CellSens Dimension v1.18 software (Olympus), for both HuARLT2 and LEC cell lines and their rKSHV-infected matches (Fig. 2).

Regions of interest (ROI) for analysis were extracted from phase contrast images and saved as separate images using FIJI. The confluency computation feature of the Phase Contrast Microscopy Segmentation Toolbox (PHANTAST) [32] plugin in FIJI was used to ensure that the extracted ROI images had comparable confluency levels.

The extracted ROI images underwent a series of pre-processing steps using FIJI. First, the images were converted to an 8-bit format to adjust brightness and contrast, enhancing cell boundary visibility. Afterwards, the images were converted to a 32-bit format to ensure compatibility with the PHANTAST plugin.

Image segmentation was performed using the PHANTAST plugin, and it involved adjusting the σ value, which controls the scale of Gaussian smoothing, and the ε value, which governs edge sensitivity or thresholding parameters for each image. Additionally, halo correction was selectively applied to enhance poorly resolved images and improve segmentation accuracy.

PHANTAST was used to generate binary masks that delineated the contours of individual cells within the images. These binary masks were then used to quantify the following cell shape parameters associated with the degree of cellular spindling using FIJI.

**Aspect ratio (AR)** is the ratio of the longest dimension to the shortest dimension in each cell to evaluate the degree of elongation of the cell,

3 $${\text{AR}}_{{{\text{cell}}}} = \frac{{\text{L}}}{{\text{W}}}$$

where L is the length of the longest dimension drawn from one end to the other, and W is the length of the shortest dimension orthogonal to L. Aspect ratio ranges from 0 to positive infinity and higher aspect ratio values indicate greater elongation and spindling.

**Roundness (R)** quantifies how closely the cell shape approximates a circle,

4 $${\text{R}}_{{{\text{cell}}}} = \frac{{4{\text{A}}}}{{\uppi {\text{D}}^{2} }}$$

where *A* is the cell’s surface area, and *D* represents the major axis of the cell passing through the centre. Roundness ranges from 0 to 1, with higher values indicating a shape that is closer to a circle. However, it does not account for the cell boundary’s irregularities.

**Circularity (C)** quantifies how closely a cell’s perimeter resembles that of a circle,

5 $${\text{C}}_{{{\text{cell}}}} = \frac{{4\uppi {\text{A}}}}{{{\text{P}}^{2} }}$$

where A is the cell’s projected area, and P is the cell’s perimeter. Like roundness, circularity ranges from 0 to 1, with higher values indicating a more circular shape. Unlike roundness, circularity considers the shape of the cell’s boundary.

### Statistical Analysis

The study aimed to determine cellular morpho-mechanical differences between uninfected vascular (HuARLT2) and lymphatic endothelial cells (LEC) and their rKSHV-infected counterparts. Statistical comparisons were performed between these biologically distinct groups.

The normality of the data was assessed using the Shapiro–Wilk test (α = 0.05). For normally or log-normally distributed data, Welch’s t-test was used to evaluate differences in intracellular viscoelasticity (bulk MSD and MSD power law exponent) and cell morphology (aspect ratio, roundness and circularity), with group statistics reported as mean ± SD. For non-normally distributed data, differences between groups were assessed using the Mann–Whitney U test, with group statistics reported as median and 95% confidence interval (Mdn [95% CI]).

Differences between groups were expressed as relative differences, with adjustments made for non-normally distributed data using Hodges-Lehmann differences. Statistical significance was considered at p < 0.05.

All experiments were conducted on two independent days, and statistical analyses were performed using Python 3.8 (Python Software Foundation, Python Language Reference, version 3.8) and GraphPad Prism 9.4.1 (GraphPad Software Inc., San Diego, CA, USA).

## Results

**Fig. 3:**
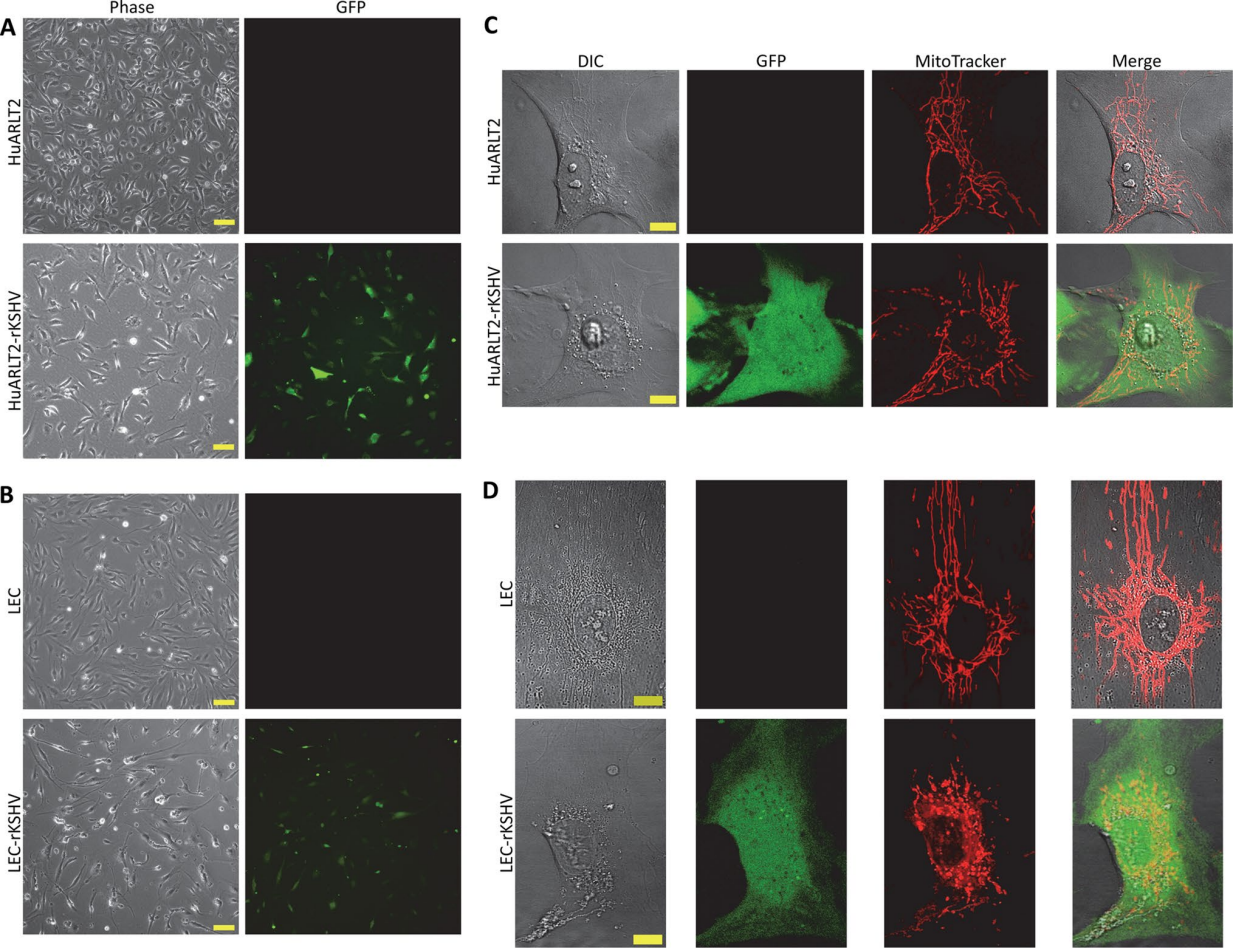
Representative micrographs of uninfected and latently infected (rKSHV) HuARLT2 and LEC cells. Phase contrast and fluorescence images of **A** HuARLT2 and HuARLT2-rKSHV cells and **B** LEC and LEC-rKSHV cells (10×, scale bar: 100 μm). Confocal images of single **C** HuARLT2 and HuARLT2-rKSHV cells and **D** LEC and LEC-rKSHV (60×, scale bar: 10 μm). GFP indicates latent rKSHV infection. MitoTracker Red fluorescently labels mitochondria for mitochondria-tracking microrheology. (DIC stands for Differential Interference Contrast imaging).

The distinctive spindle phenotype is a hallmark signature in KSHV-infected primary endothelial cells. The present study quantified the morphological changes associated with spindling in latently rKSHV-infected HuARLT2 and LEC cells, as well as changes in intracellular mechanical properties for use as potential physical biomarkers. Stably KSHV-infected HuARLT2 and LEC cells were identified by GFP expression as a marker for rKSHV latent infection (see section “Cell Lines”). These cells and uninfected controls were fluorescently labelled with MitoTracker Red for mitochondria-tracking microrheology analysis (Fig. 3).

### rKSHV.219 Establishes Latency and Induces Morphological Changes in HuARLT2 and LEC

**Fig. 4:**
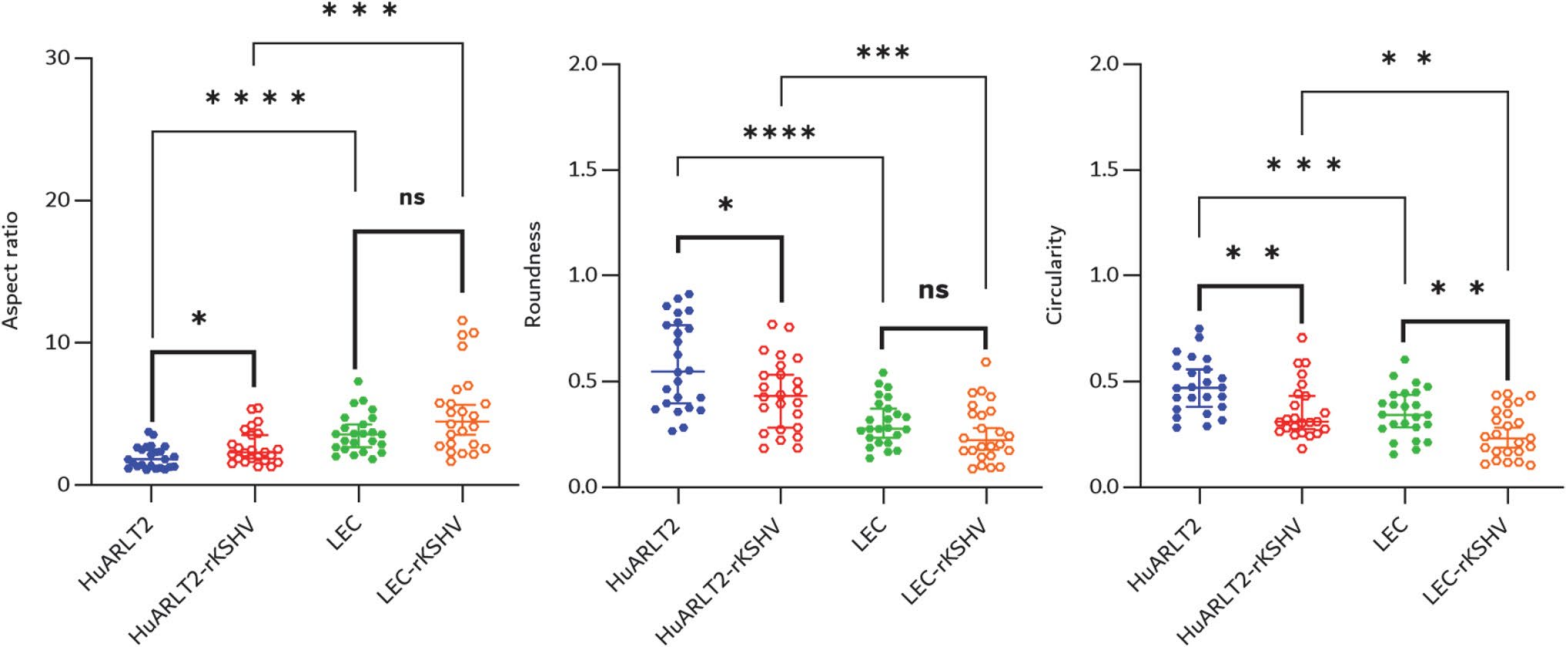
Cellular morphology. HuARLT2-rKSHV exhibit a significantly higher aspect ratio, lower roundness, and lower circularity than uninfected HuARLT2. Similarly, LEC-rKSHV display increased aspect ratio, lower roundness, and significantly lower circularity compared to uninfected LEC. LEC display stronger spindloid features than HuARLT2 both in the uninfected and the infected state. N = 24 for each case, and each data point represents measurements from a single cell. Plots represent mean ± standard deviation for normally distributed data and median and 95% confidence interval for not normally distributed data. (*p < 0.05, **p < 0.01, ***p < 0.001, ****p < 0.0001, *ns* non-significant.)

Comparing HuARLT2-rKSHV and LEC-rKSHV with their uninfected controls, cell morphology analyses revealed considerably higher elongation in infected cells as quantified by cell aspect ratio (A), roundness (R), and circularity (C) (Fig. 4). HuARLT2-rKSHV exhibited a significantly higher aspect ratio (Mdn 2.315 [1.879, 3.532] vs 1.825 [1.306, 2.517], p = 0.0271), lower roundness (Mdn 0.432 [0.283, 0.532] vs 0.548 [0.397, 0.765], p = 0.0271), and lower circularity (Mdn 0.310 [0.276, 0.432] vs 0.472 [0.380, 0.557], p = 0.0022) than HuARLT2. Similarly, LEC-rKSHV displayed an increased aspect ratio (4.605 ± 2.914 vs 3.600 ± 1.396, p = 0.106), decreased roundness (0.257 ± 0.136 vs 0.308 ± 0.110, p = 0.152), and decreased circularity (0.256 ± 0.115 vs 0.352 ± 0.116, p = 0.0058) than the uninfected LEC, although only the change in circularity was significant.

When comparing uninfected HuARLT2 and LEC cells, the aspect ratio was significantly higher in LECs (Mdn 1.825 [1.306, 2.517] vs. 3.600 [2.691, 4.281], p < 0.0001), whereas roundness (0.583 ± 0.210 vs. 0.308 ± 0.110, p < 0.0001) and circularity (0.477 ± 0.129 vs. 0.352 ± 0.116, p = 0.0009) were significantly lower in LECs. Similarly, when comparing HuARLT2-rKSHV to LEC-rKSHV, LEC-rKSHV displayed a greater aspect ratio (Mdn 2.315 [1.879, 3.532] vs. 4.605 [2.879, 5.794], p = 0.0002), along with significantly lower roundness (0.583 ± 0.210 vs. 0.308 ± 0.110, p = 0.0002) and circularity (0.310 [0.276, 0.432] vs. 0.237 [0.169, 0.349], p = 0.0073) (Fig. 4).

In essence, rKSHV-infected HuARLT2 cells showed a significant 29.7% increase in aspect ratio, along with a 26.1% reduction in roundness and a 25.7% decrease in circularity compared to uninfected controls. In contrast, rKSHV-infected LECs showed a smaller, statistically insignificant 26.0% increase in aspect ratio, a 16.9% decrease in roundness, and a significant 27.3% decrease in circularity compared to their uninfected counterparts. Latent rKSHV infection increased the aspect ratio and decreased roundness and circularity for both HuARLT2s and LECs, although the changes for roundness and circularity are not significant for LECs.

### Increased MSD Amplitude and Decreased MSD Power Law Exponent are Observed with rKSHV-Induced Cellular Elongation

**Fig. 5:**
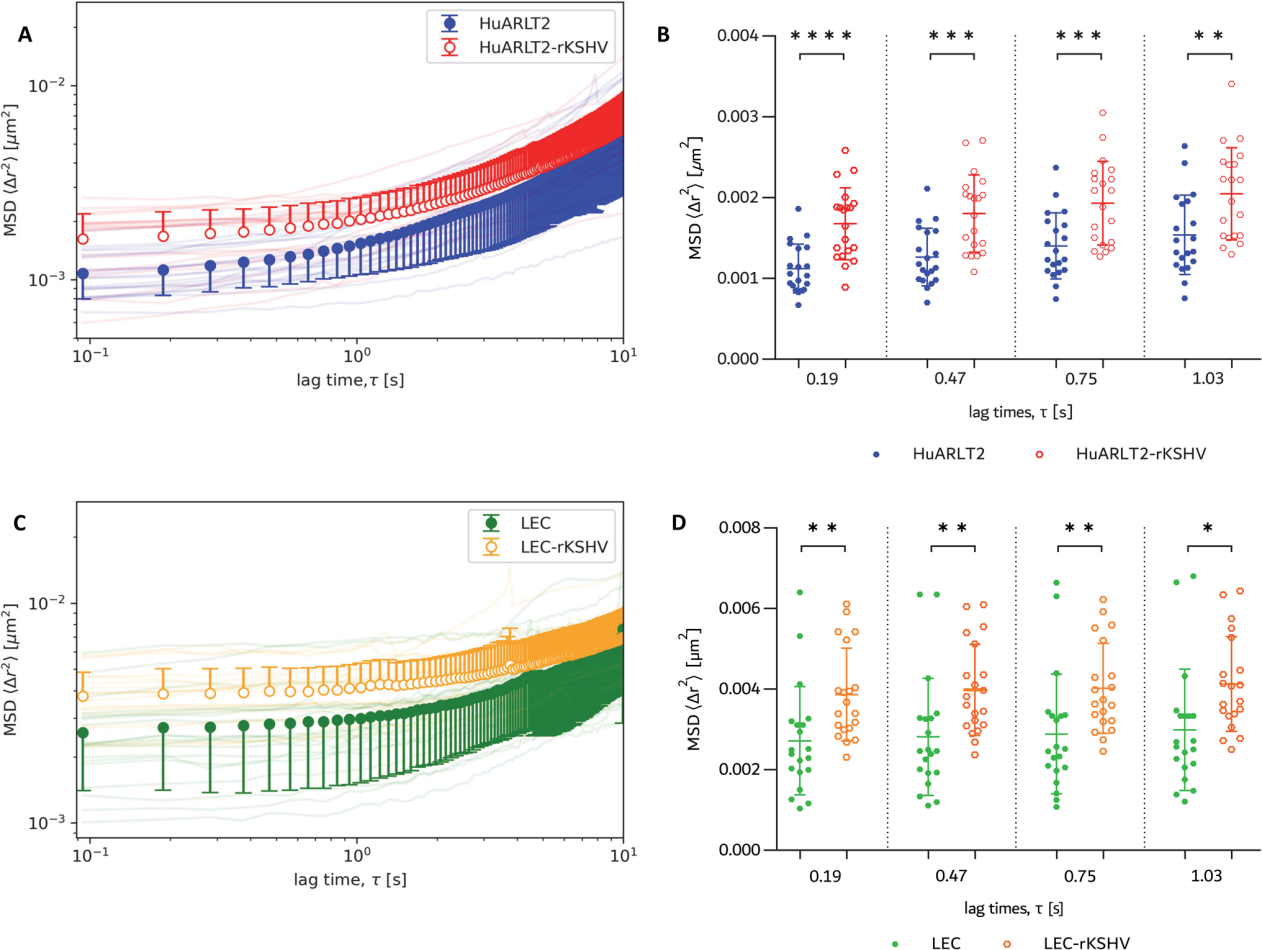
Mitochondria-tracking microrheology results of HuARLT2 (**A**, **B**) and LEC (**C**, **D**). **A**, **C** Bulk MSD for uninfected (solid circles) and infected cells (open circles) for a lag time of 0 s ≤ τ ≤ 10 s. Error bars represent the standard deviation (SD). Ensemble-averaged MSD of individual cells are shown as faint lines. **B**, **D** Ensemble-averaged MSD for individual cells and bulk MSD (mean ± SD) for lag times of 0 s ≤ τ < ≈ 1 s. *p < 0.05, **p < 0.001, ***p < 0.0001, ****p < 0.00001 (N = 20). Each data point represents measurements from a single cell

The latently infected cells portrayed significantly higher mitochondrial MSD amplitudes than uninfected cells, with differences decreasing with increasing lag time (Fig. 5A, C). For short lag times between τ = 0 s and τ ≈ 1 s with predominantly passive mitochondrial fluctuations, the MSD amplitude of HuARLT2-rKSHV was 49.4% higher (p = 0.0001) at τ = 0.19 s and 33.0% higher (p = 0.0046) at τ = 1.03 s than that of HuARLT2 (Fig. 5B). The MSD amplitudes of LEC-rKSHV were between 42.2% (p = 0.0062, τ = 0.19 s) and 38.0% (p = 0.0115, τ = 1.03 s) higher than for LEC (Fig. 5D).

**Fig. 6:**
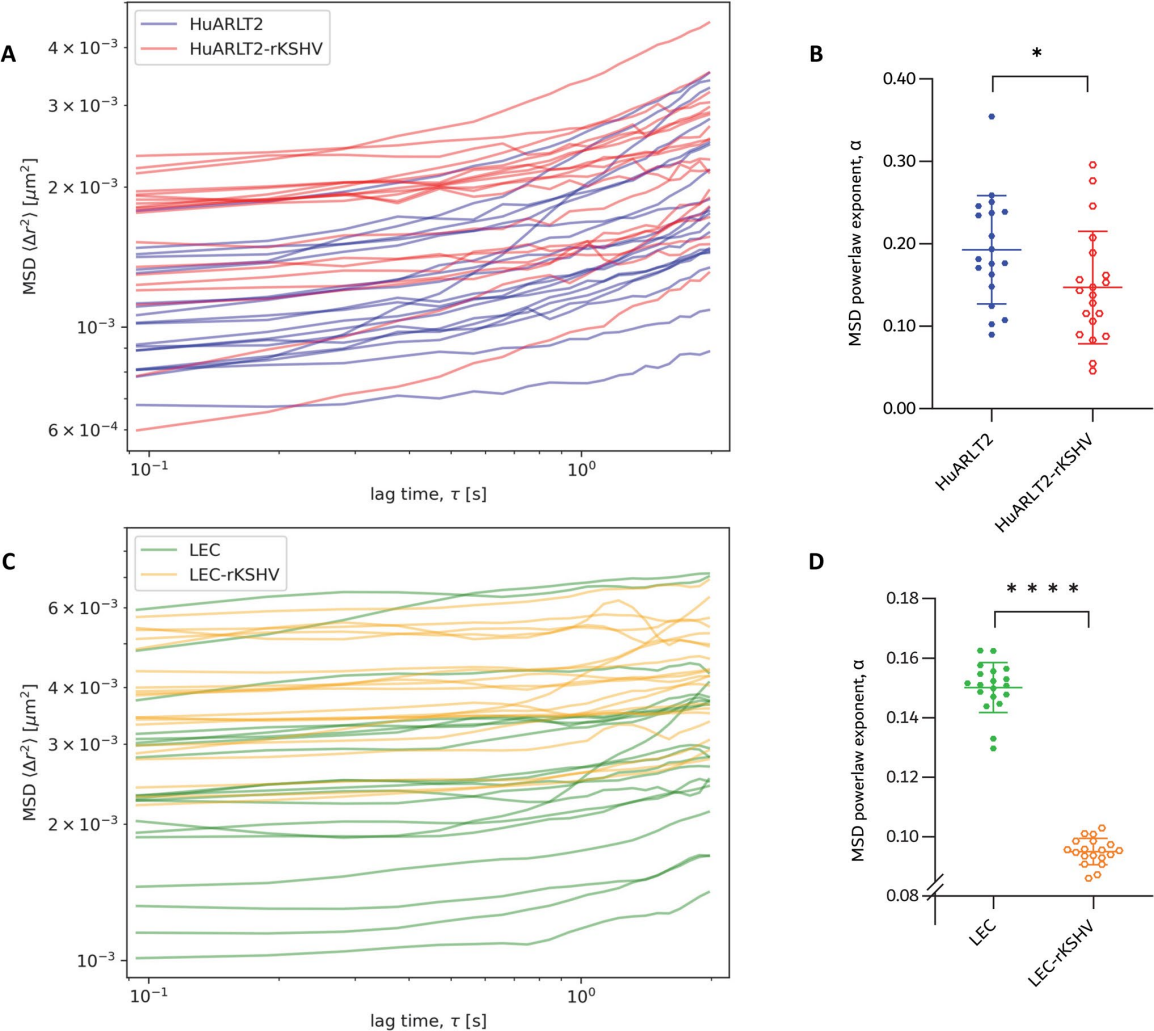
MSD and MSD power law exponent α HuARLT2 (**A**, **B**) and LEC (**C**, **D**) for a lag time of 0 s ≤ τ ≤ 2 s. Individual cell ensemble-averaged MSD curves (**A**, **C**) and fitted MSD power law exponent α (**B**, **D**) for uninfected (blue and green, solid markers) and infected cells (red and orange, open markers) for 0 s ≤ τ ≤ 2 s. Each data point represents measurements from a single cell. The Savitzky–Golay filter was used with W_SG_ = 7 and P_SG_ = 3 to smoothen MSD curves for improved curve fitting for LEC-rKSHV and LEC (C)**.** *p < 0.05, **p < 0.001, ***p < 0.0001, ****p < 0.00001

When fitted to a power law function according to the behaviour of particles diffusing in a viscoelastic medium [Eq. (2)], the infected cells (HuARLT2-rKSHV and LEC-rKSHV) exhibited significantly lower MSD power law exponent α than the uninfected counterparts (HuARLT2 and LEC) over a short lag time range of 0 s ≤ τ ≤ 2 s (23.9%, p = 0.0395 and 36.7%, p = 0.0001, respectively) (Fig. 6).

## Discussion

The present study investigated changes in cellular morphology and viscoelasticity induced by rKSHV infection in HuARLT2 and LEC cells to identify potential morpho-mechanical markers of infection and the early-stages of KS tumorigenesis.

The spindle phenotype in KSHV-infected endothelial cells is a crucial diagnostic parameter in KS histopathology; however, it has only been reported qualitatively to date [33]. Accurate histopathological diagnosis of KS spindle cells relies heavily on pathologist expertise, especially in the early stages of the disease, as other malignancies share a similar morphological profile [5, 34]. Identifying latently infected cells poses challenges due to the conservative gene expression profile of KSHV during latency [4]. Consequently, quantifying morphological changes is a key step towards robustly detecting infection in endothelial cells.

In the present study, relative to uninfected controls, HuARLT2-rKSHV cells displayed an approximately 1.3 times higher aspect ratio and about 0.74 times lower roundness and circularity, indicating marked transformation to a spindloid phenotype associated with rKSHV infection. The extent of spindling in LEC-rKSHV was lower than in HuARLT2-rKSHV, with an aspect ratio approximately 1.26 times greater than that of uninfected LEC and a reduction in roundness to about 0.83 times that of uninfected LECs. The reduced spindling observed in LEC-rKSHV was likely due to their pre-existing elongated morphology, as uninfected LECs already exhibit a more spindle-shaped phenotype compared to uninfected HuARLT2 cells (Fig. 4). This is consistent with previous with previous reports which showed that primary LECs tend to have a more elongated morphology than vascular ECs when grown and maintained under 2D in vitro conditions [35]. Therefore, the aspect ratio and roundness measurements may not be sensitive enough to confidently detect morphological changes associated with rKSHV infection in LECs. However, the observed decrease in circularity of rKSHV-infected LECs to approximately 0.73 times that of uninfected controls supports the presence of spindling (Fig. 4). This quantification of spindling in rKSHV-infected HuARLT2 and LECs, known to be associated with actin cytoskeleton reorganisation [35], represents a significant step in identifying EC morphological changes that are indicative of herpesvirus infection in endothelial cells of both vascular and lymphatic origin. Although in the present study, the PHANTAST plugin which includes halo adjustment step to mitigate segmentation and boundary artifacts was used, minor segmentation errors may impact the accuracy of cell morphology calculations. However, since all conditions were processed using the same pipeline, the relative differences observed between uninfected and infected cells remain valid (Fig. 4). Future studies can benefit from a combination of higher-resolution imaging approaches and alternative segmentation tools such as deep learning-based contour detection.

From a diagnostic perspective, these findings may suggest a potential limitation of cell shape-based diagnostics to detect KSHV infection. While morphological changes in vascular endothelial cells (for example, HuALRT2) are more pronounced and readily detectable, the pre-existing elongated morphology of LECs reduces the sensitivity of such approaches. This highlights the need to thoroughly consider cell type-specific baseline morphology when developing diagnostic tools based on morphological changes.

The MSD amplitude of tracer particles is directly proportional to the local intracellular deformability at a given lag time, τ [36]. In the current study, HuARLT2-rKSHV and LEC-rKSHV showed 40.6% ± 7.0% (p < 0.0015) and 40.2% ± 1.9% (p < 0.0088) higher MSD amplitudes, respectively, than the uninfected controls for Brownian motion-dominated lag times of 0 < τ ≤ 1.03 s. This increase in MSD amplitude, therefore, suggests a decrease in local cellular stiffness, which coincides with the vFLIP-mediated spindling and altered actin cytoskeleton architecture. While the precise link between these MSD differences and their molecular aetiology remains unclear, the distinct upward shift in MSD amplitudes of infected cells enables clear distinction from uninfected cell populations. Based on this result, rKSHV infection plausibly increases the mesh size between actin filaments, i.e., inter-filament distance, which then contributes to the more deformable phenotype observed in infected cells. The morphological analyses in the present study align with previous reports of significant actin cytoskeleton reorganisation in infected cells [4], which may result in a less dense and more flexible actin network consistent with a deformable cell mechanical phenotype. While viral infections are known to modulate host cell cytoskeletal dynamics [37, 38], direct evidence linking rKSHV infection to specific changes in actin filament organisation is limited. Therefore, further molecular analyses of actin cytoskeletal components will be necessary to substantiate the proposed hypothesis.

The mechanotyping of cancer cells can be inherently complex and depends on factors such as the specific malignancy and the analytical method and equipment used [39]. It is important to acknowledge that, in the present study, all experiments were conducted under 2D in vitro culture conditions on tissue culture plastic, which is significantly stiffer than physiological tissues. The ability of cells to actively sense and respond to substrate rigidity can influence their mechanical properties [40]. However, because both infected and uninfected cells were cultured under identical conditions, the relative changes in mechanical properties induced by rKSHV infection remain physiologically meaningful. The results suggest that these changes primarily result from intracellular remodelling driven by viral infection rather than substrate stiffness alone. Future studies could explore in vivo infection models to assess how endothelial cell mechanics are altered in a more physiologically relevant microenvironment. Also, the MSD amplitude may be insufficient to characterise the viscoelasticity of the cytoplasm fully [41]. However, in the current study, the MSD effectively captures the difference in deformability of the cells related to the presumably reorganised actin cytoskeleton of infected HuARLT2 and LEC cells compared to uninfected controls. The results reported here may be a unique phenomenon observed only within these specific cell lines, with the specific recombinant KSHV strain used, and at the specific time point post-infection.

All values of the MSD power law exponent, α, across the cell lines were below 0.5, confirming the expected viscoelastic nature of the intracellular environment and constrained mitochondrial motion for both infected and uninfected cells [42]. This indicates that mitochondrial fluctuations were predominantly passive and driven by thermal processes. The α value was 23.9% (p < 0.0395) and 36.7% (p < 0.0001) lower in the HuARLT2-rKSHV and LEC-rKSHV, respectively than in the associated uninfected controls for lag times of 0 < τ ≤ 2 s. The significant decrease in the power law exponent suggests a more viscous-like cytosol [43] in the infected than the uninfected cells. Despite higher MSD amplitudes, which suggest a larger actin cytoskeleton mesh size, the cytosol in infected cells is less permissive of mitochondrial fluctuations. The observed α values indicate alterations in the diffusive behaviour of mitochondria within the cells, and the decrease in α values may signify a shift towards more constrained or hindered mitochondrial motion, expected with an increase in cytosolic viscosity. The interpretation of the α value is multifaceted, influenced by factors such as cytoskeletal organisation, molecular interactions, and cellular microenvironment. Studies have shown that the MSD and its power law exponent are not always concordant [41]. The increased MSD values observed in the current study point to increased bulk deformability of the cytoplasm, likely due to actin reorganisation that allows for wider mitochondrial excursions post infection. In contrast, the decrease in α values, which indicates the diffusive mode of tracked mitochondria, may reflect increased molecular crowding that effectively increases cytoplasmic microviscosity. Future studies could incorporate complementary techniques, such as active microrheology or fluorescence recovery after photobleaching (FRAP), to further validate the current findings.

The morphological and mechanical findings presented here suggest that the documented actin cytoskeleton reorganisation in endothelial cells during the early stages of KSHV infection [3] is coupled with distinct and sustained changes in cellular morphology and viscoelasticity.

Interestingly, morphological changes of cells associated with spindling due to rKSHV infection were smaller in LEC than in HuARLT2 cells. In contrast, changes in mechanical properties (MSD and α) were the same in both cell lines if not higher in LEC than HuARLT2. This finding may suggest that intracellular mechanics may be a more sensitive discriminator than cell morphology for detecting rKSHV infection in endothelial cell lines.

It is acknowledged that cellular morphology in a 2D monolayer differs from that observed in histopathological tissue sections due to the absence of extracellular matrix (ECM) constraints. In vivo, endothelial cells interact with the basement membrane and neighbouring cells in a mechanically complex 3D microenvironment, which influences their morphology. However, 2D cultures still allow for precise quantification of infection-induced changes independent of these external forces. To bridge the gap between in vitro and in vivo conditions, future studies incorporating 3D models—such as ECM-coated substrates and shear flow experiments—can provide a more physiologically relevant context. Direct comparisons with histopathological analyses can also help validate these findings. While dimensionality and ECM constraints contribute to morphological differences, the primary focus of the current study was on quantifying relative morphological changes rather than isolating their specific causes.

Previous studies using active and passive microrheology have distinguished malignant from premalignant or benign cells in cancers like melanoma [44], ovarian cancer [45], and breast cancer [46]. While some cancer cells stiffen [42], most become more deformable driven by cytoskeletal remodelling [47]. Passive microrheology, using bead- and mitochondria-tracking microrheology (MTM), has shown that cytoskeletal disruptions can affect cellular viscoelasticity [24, 25]. Although studies on virus-induced mechanical changes are limited, cytoskeletal reorganisation is a well-established consequence of KSHV infection [4]. Since the cytoskeleton governs intracellular viscoelasticity, the actin reorganisation resulting from infection likely underlies the differences observed in our study. The findings of the current study contribute to this growing body of work by demonstrating that KSHV infection induces distinct biophysical changes in endothelial cells in the form of increased deformability (higher MSD amplitude) and increased cytosolic viscosity (lower MSD power law exponent). Given that KS is a multifocal tumour with low metastatic potential [48], the increased cellular deformability observed post-infection may contribute to the lymphatic leakiness and oedema characteristic of late-stage KS [49].

## Conclusion

During KS tumorigenesis, the infection of endothelial cells by KSHV is deemed necessary though insufficient to result in the malignant transformation of endothelial cells. While KSHV infection initiates the process of oncogenesis in endothelial cells, the development of KS only occurs when specific complementary conditions, such as immunosuppression and inflammation, are also present. It remains crucial, however, to detect KSHV infection as early as possible.

The current study demonstrates the cellular morphological and mechanical changes induced by rKSHV infection in HuARLT2s and LECs as a model for understanding the early phases of KS tumorigenesis. The results indicate that the cytoskeletal reorganisation known to result from KSHV infection of endothelial cells is associated with noticeable changes in cellular morphology and viscoelastic properties. While changes in cell morphology have been qualitatively observed through conventional histopathological staining in KS diagnosis, investigations into intracellular mechanics associated with this transformation have been lacking. The findings of the present study suggest that rKSHV infection leads to increased cell deformability, which may not only serve as a marker of early KS malignancy but also play a role in the pathogenesis of KS. Notably, increased cellular deformability following infection is observed in ECs of both vascular and lymphatic lineages that are implicated as primary targets of KSHV during KS tumorigenesis.

The present study underscores the significant role of a biophysical approach in elucidating virus-mediated oncogenesis. The findings contribute to understanding virus-mediated oncogenesis and developing novel strategies for early diagnosis of KSHV-associated malignancies. Augmentation with low-cost, automated, high-throughput microfluidics systems offers potential for point-of-care applications in under-resourced settings following the validation of physical cell markers of malignancy.

## Data Availability

Data supporting this study are available on the University of Cape Town’s institutional data repository ZivaHub under the https://doi.org/10.25375/uct.27043813 as Shabangu MM, Blumenthal MJ, Sass DT, Lang DM, Schafer G, Franz T. Data and software code for “Endothelial cells stably infected with recombinant Kaposi’s sarcoma-associated herpesvirus display distinct viscoelastic and morphological properties”. ZivaHub, 2024, https://doi.org/10.25375/uct.27043813.
